# Fucoxanthin prevents breast cancer metastasis by interrupting circulating tumor cells adhesion and transendothelial migration

**DOI:** 10.3389/fphar.2022.960375

**Published:** 2022-09-09

**Authors:** Weiyu Wang, Chengbin Fu, Mengting Lin, Yusheng Lu, Shu Lian, Xiaodong Xie, Guiyu Zhou, Wulin Li, Yiping Zhang, Lee Jia, Chunlian Zhong, Mingqing Huang

**Affiliations:** ^1^ Fujian Key Laboratory of Chinese Materia Medica, College of Pharmacy, Fujian University of Traditional Chinese Medicine, Fuzhou, Fujian, China; ^2^ Fujian-Taiwan-Hongkong-Macao Science and Technology Cooperation Base of Intelligent Pharmaceutics, College of Material and Chemical Engineering, Minjiang University, Fuzhou, Fujian, China; ^3^ Fujian Key Laboratory on Conservation and Sustainable Utilization of Marine Biodiversity, Fuzhou Institute of Oceanography, Minjiang University, Fuzhou, Fujian, China; ^4^ Department of Breast Surgery, Fujian Medical University Union Hospital, Fuzhou, China; ^5^ Breast Surgery Institute, Fujian Medical University, Fuzhou, China; ^6^ College of Chemistry, Fuzhou University, Fuzhou, China; ^7^ Technical Innovation Center for Utilization of Marine Biological Resources, Third Institute of Oceanography, Ministry of Natural Resources, Xiamen, China

**Keywords:** fucoxanthin (CID: 5281239), cancer metastasis chemoprevention, transendothelial migration, cell adhesion, epithelial-to-mesenchymal transition (EMT), NF-κB signaling pathway, PI3K/Akt signaling pathway, FAK/Paxillin signaling pathway

## Abstract

Metastasis is the leading cause of cancer-related death and a critical challenge in improving cancer treatment today. Circulating tumor cells (CTCs) adhesion to and across the vascular endothelium are critical steps in the establishment of micrometastatic foci away from the primary tumor. Therefore, we believe that interrupting CTCs adhesion to endothelium and transendothelial migration may efficiently prevent cancer metastasis. Fucoxanthin (Fx) is an algal carotenoid widely distributed in brown algae, macroalgae, and diatoms. Previous studies have found that Fx has various pharmacological activities, including antidiabetic, antioxidant, anti-inflammatory, anti-obesity, antimalarial, anticancer, and so on. However, it remains unclear whether Fx has a preventive effect on cancer metastasis. Here, we found that Fx interrupts breast cancer cells MCF-7 adhesion to endothelium and transendothelial migration, thus inhibiting CTCs-based pulmonary metastasis *in vivo*. The hetero-adhesion assay showed that Fx significantly inhibited the expression of inflammatory factor-induced cell adhesion molecules (CAMs) and the resulting adhesion between MCF-7 cells and endothelial cells. The wound-healing and transwell assays showed that Fx significantly inhibited the motility, invasion, and transendothelial migration abilities of MCF-7 cells. However, the same concentration of Fx did not significantly alter the cell viability, cell cycle, apoptosis, and ROS of breast cancer cells, thus excluding the possibility that Fx inhibits MCF-7 cell adhesion and transendothelial migration through cytotoxicity. Mechanistically, Fx inhibits the expression of CAMs on endothelial cells by inhibiting the NF-кB signaling pathway by down-regulating the phosphorylation level of IKK-α/β, IкB-α, and NF-кB p65. Fx inhibits transendothelial migration of MCF-7 cells by inhibiting Epithelial-to-mesenchymal transition (EMT), PI3K/AKT, and FAK/Paxillin signaling pathways. Moreover, we demonstrated that Fx significantly inhibits the formation of lung micrometastatic foci in immunocompetent syngeneic mouse breast cancer metastasis models. We also showed that Fx enhances antitumor immune responses by substantially increasing the subsets of cytotoxic T lymphocytes in the peripheral immune system. This new finding provides a basis for the application of Fx in cancer metastatic chemoprevention and suggests that interruption of the CTCs adhesion to endothelium and transendothelial migration may serve as a new avenue for cancer metastatic chemoprevention.

## Introduction

Cancer is a serious global public health problem, about 19.3 million new cancer diagnoses and 10.0 million cancer-related deaths occurred in 2020 worldwide (Bray et al., 2021; Sung et al., 2021; Tan et al., 2021). Metastasis is the leading cause of cancer-related death and a critical clinical obstacle to cancer treatment (Sethi and Kang, 2011). Breast cancer is the most frequently diagnosed cancer in women, and over 90% of cancer-related deaths in breast cancer are caused by cancer recurrence and metastasis (Chen et al., 2018; van Veen et al., 2018). Despite advances in diagnostic techniques and treatments for cancer in recent years, the prognosis of most cancer survivors is still unsatisfactory due to metastasis. Therefore, it is necessary to explore a new strategy to prevent cancer metastasis during and after the surgical removal of primary tumors (Lu et al., 2019a).

Cancer metastasis is an extremely complex process, and circulating tumor cells (CTCs) entering the bloodstream microenvironment must successfully complete a series of sequential steps to metastasize to distant organs through hematogenous dissemination (Lu et al., 2019a; Lu et al., 2019b). The adhesion and transendothelial migration of CTCs in the bloodstream microenvironment are critical steps in the establishment of distant micrometastatic foci. Because CTCs in the bloodstream microenvironment generally exhibit a low rate of proliferation, current chemotherapeutic agents originally designed to target highly proliferating cancer cells are not only ineffective in killing CTCs but may cause intolerable side effects by destroying proliferating non-cancer cells (Meng et al., 2004; Stott et al., 2010). Therefore, chemotherapeutic agents cannot be used to prevent cancer metastasis in asymptomatic cancer survivors. In fact, anticancer chemotherapy sometimes promotes the formation of metastases (Daenen et al., 2011). We need to develop a more effective and nontoxicity method to prevent cancer metastasis.

Extensive evidence indicates that if CTCs do not successfully adhere to the vascular endothelium, they may trigger apoptotic death through blood shear stress, a process referred to as anoikis (Chen et al., 2013; Wan et al., 2015; Luey and May 2016). Furthermore, since CTCs in the bloodstream microenvironment are extremely vulnerable to immune cells, they must migrate across endothelial cells to metastatic sites as quickly as possible (Labelle and Hynes, 2012). Cell adhesion molecules (CAMs) expressed on the surface of endothelial cells play a critical role in attracting CTCs to adhere to the vascular endothelium, and the expression of CAMs including VCAM-1, ICAM-1, and E-selectin is regulated by inflammatory factor-induced NF-кB signaling pathway (Hoesel and Schmid, 2013; Lu et al., 2014; Lian et al., 2016; Lu et al., 2019a). Transendothelial migration of CTCs is similar to the pathway used by leukocytes during inflammation (Reymond et al., 2013; Mannion et al., 2021). Regulatory molecules involved in the transendothelial migration of leukocytes and cancer cells could serve as potential targets for pharmaceutical compound screening (Salminen et al., 2020). Epithelial-to-mesenchymal transition (EMT) and motility of cancer cells are associated with transendothelial migration, and these processes are regulated by the PI3K/AKT and FAK/Paxillin pathways (Xue and Hemmings, 2013; Lamouille et al., 2014; Yu et al., 2015). Therefore, based on the above understanding of the process of cancer metastasis, we proposed that inhibition of CTCs adhesion and transendothelial migration may effectivity prevent cancer metastasis.

Fucoxanthin (Fx) is an algal marine carotenoid widely distributed in brown algae, macroalgae, and diatoms (Peng et al., 2011). Previous studies have found that Fx has various pharmacological activities, including antidiabetic, antioxidant, anti-inflammatory, anti-obesity, antimalarial, anticancer, and so on (Maeda et al., 2007; Rodriguez-Luna et al., 2019; Meresse et al., 2020). Since regular consumption of seaweed (rich in fucoxanthin) increases longevity (Sho, 2001), we speculated that Fx may have a role in preventing tumor metastasis. In recent years, more and more studies showed that Fx has anti-cancer activities by inhibiting cell proliferation and inducing apoptosis (Kumar et al., 2013; Rwigemera et al., 2015; Meresse et al., 2020; Ming et al., 2021). Although Fx has several biological and biochemical activities, the effects and regulatory mechanisms of low-dose Fx (weakly cytotoxicity) in prevent cancer metastasis remains unclear.

In the current study, we investigated the potential and molecular mechanisms of low-dose Fx (weakly cytotoxicity) to prevent cancer metastasis both *in vitro* and *in vivo*. We report the Fx *in vitro* effects on cell viability, hetero-adhesion, invasion, transendothelial migration, and expression of CAMs. In addition, we investigated the molecular mechanisms by which Fx inhibits cancer cells adhesion and transendothelial migration. We also performed *in vivo* experiments to analyze the cancer metastasis prevention effect of Fx on the syngeneic mouse experiment metastatic models.

## Materials and methods

### Cell culture

Human breast cancer cells MCF-7 and mouse breast cancer cells 4T1 were obtained from the Cell Bank of the Chinese Academy (Shanghai, China). MCF-7 and 4T1 cells were cultured in Dulbecco’s modified Eagle’s medium (DMEM) high-glucose medium (Hyclone) supplemented with 10% fetal bovine serum (FBS, obtained from Gibico), 100 U/ml penicillin and 100 μg/ml streptomycin. Human umbilical vein endothelial cells (HUVECs) were isolated from human umbilical veins asdescribed previously (Lu et al., 2014; Lu et al., 2019a), and cells were cultured in endothelial cell medium (ECM, obtained from ScienCell) supplemented with 5% FBS, 100 μg/ml endothelial cell growth supplement (ECGS, obtained from ScienCell), 100 U/ml penicillin and 100 μg/ml streptomycin. All cells were maintained at 37°C in a humidified atmosphere of 5% CO_2_ and harvested with 0.25% trypsin (GenView).

### Cell viability assay

The cytostatic effect of Fx was determined by using the cell counting kit 8 (CCK8) as previously described (Lu et al., 2014; Lu et al., 2019a; Zhong et al., 2020). Cells (1 × 10^4^/well) were seeded in 96-well plates and cultured for 24 h. Then the cells were treated with the medium containing various concentrations of Fx (0 μM, 5 μM, 10 μM, 20 μM, 30 μM, 40 μM, 50 μM, 60 μM, and 70 μM) for 24 h. After 24 h incubation, 10-μl of CCK8 was added to the cells and the incubation was continued for 1 h at 37°C. Cell viability of cells was determined by measuring the absorbance at 450 nm in a multifunctional microplate reader (TECAN, M200 PRO), and the results were expressed as the relative ratio of cell viability compared to the untreated control.

### Cell cycle assay

The cell cycle was determined by flow cytometry using the cell cycle analysis kit (Lu et al., 2014; Zhong et al., 2020). Cells were seeded at a density of 1 × 10^5^ cells per well in a six-well plate for 24 h. Then the cells were treated with various concentrations of Fx (0 μM, 1 μM, 5 μM, 10 μM, 25 μM, and 50 μM) for 24 h. The cells were washed with ice-cold PBS and fixed with 1 ml of cold 75% ethanol for overnight at 4°C. After that, the cells were washed with PBS and stained with PI staining working solution. The cells were incubated for 45 min at room temperature in the dark. The cell cycle was detected by flow cytometry (BD, FACSAriaIII) and analyzed by FlowJo software.

### Cell apoptosis assay

Cell apoptosis was determined by flow cytometry using the annexin V-FITC/PI staining kit (Cheng et al., 2018; Zhong et al., 2020). Cells were seeded at a density of 1 × 10^5^ cells per well in a six-well plate for 24 h. Then the cells were treated with various concentrations of Fx (0 μM, 1 μM, 5 μM, 10 μM, 25 μM, and 50 μM) for another 24 h. The cells were washed with PBS and resuspended in binding buffer, followed by staining with Annexin V-FITC and PI for 15 min in the dark. The cell apoptosis was assessed by flow cytometry and analyzed by FlowJo software.

### Intracellular reactive oxygen species detection

The intracellular ROS was detected by flow cytometry using the reactive oxygen species assay kit (Beyotime). MCF-7 cells were treated with various concentrations of Fx (0 μM, 1 μM, 5 μM, 10 μM, 25 μM, and 50 μM) for 24 h. Then the cells were washed with PBS two times, DCFH-DA was added to the culture medium, and the cells were co-incubation for another 2 h. Followed by the cells were washed with PBS two times to remove excess DCFH-DA, and resuspended in 500 μl of PBS. Intracellular ROS was detected by flow cytometry and analyzed by FlowJo software.

### Hetero-adhesion analysis of cancer cells to endothelial cells

The effects of Fx on the hetero-adhesion of MCF-7 cells to HUVECs whereas evaluated as previously described (Lu et al., 2014; Lian et al., 2016; Lu et al., 2019a; Zhong et al., 2020). Briefly, HUVECs grown to confluence in 24-well plates were pretreated with Fx (0 μM, 1 μM, 5 μM, 10 μM, and 25 μM) for 24 h, followed by the addition of TNF-α (10 ng/ml) to stimulate the HUVECs for 4 h. Fx and Rhodamine 123-labeled MCF-7 cells were added to the HUVECs monolayers and co-cultured for 1 h. After incubation, the wells were washed with PBS and non-adhered MCF-7 cells were removed. MCF-7 cells (labeled with Rhodamine 123) adhered to the endothelial cells and were photographed using a fluorescence microscope (Leica, Germany). A comparison of the number of adhered cancer cells in the treated group and control group was used to calculate the inhibition of adhesion.

### Migration assay

The effects of Fx on the migration ability of MCF-7 cells were determined by the wound-healing migration assay (Cheng et al., 2018; Zhong et al., 2020). MCF-7 cells were seeded into a 24-well plate. After the formation of cell monolayers, the cells were scratched gently by using a sterile pipette tip of 10 μl, which were washed three times with PBS and cultivated a in serum-free medium containing different concentrations of Fx (0 μM, 5 μM, 10 μM, and 25 μM). The same wounded areas were imaged at different time points (0 and 24 h, respectively) and quantified using Fiji Image J software.A comparison of the initial area and the final area of the marked areas were used to evaluate the cell migration ability.

### Invasion assay

The inhibitory effect of Fx on the invasive ability of MCF-7 cells was analyzed by the transwell invasion assay. Briefly, the transwell culture chambers (24-well, 8 μm pore size, Costar, Corning Incorporated, United States) were placed in a 24-well plate and the upper chambers were coated with Matrigel and air-dried. After 24 h, 5 × 10^4^ MCF-7 cells were suspended in 500 μl of serum-free medium containing various concentrations of Fx (0 μM, 5 μM, 10 μM, and 25 μM) and placed into the upper chamber of the wells. 500 μl of medium containing 20% FBS and the same concentration of Fx was added to the lower chambers. After a further 24 h of incubation at 37°C in 5% CO_2_, the cells in the upper chambers were carefully wiped with a cotton swab and washed with PBS. Cells that invaded the basolateral side of the upper chambers were fixed with 4% (w/v) paraformaldehyde and stained with crystal violet. The invading cells were counted and photographed using an optical microscope (Leica, Germany).

### Transendothelial migration assay

A transendothelial migration assay was performed to detect the MCF-7 cells that invaded through HUVEC monolayers without or with Fx treatment (Zhang et al., 2020). Briefly, the transwell culture chambers (24-well, 8 μm pore size, Costar, Corning, United Statws) were placed in a 24-well plate, the upper chambers were coated with Matrigel (Corning, United Statws, Cat. #356234) on the bottom and allowed to air-dry under sterile conditions. HUVECs (1 × 10^5^/well) were seeded in the upper chamber and allowed to grow to confluence. Then, the addition of TNF-α (10 ng/ml) to stimulate the monolayer of HUVECs for 4 h. The medium was removed, and GFP-labeled MCF-7 cells (5 × 10^4^/well) were suspended in 500 μl of serum-free medium containing various concentrations of Fx (0 μM, 5 μM, 10 μM, and 25 μM) and placed into the upper chamber of the wells. 500 μl of medium containing 20% FBS and the same concentration of Fx was added to the lower chambers. After a further 24 h of incubation at 37°C in 5% CO_2_, the cells in the upper chambers were carefully wiped with a cotton swab and washed with PBS. The fluorescence signal of the MCF-7 cells (labeled with GFP) that transendothelial migrated to the basolateral side of the Matrigel coated-transwell membrane was recorded by using a fluorescence microscope (Leica, Germany).

### Effect of Fucoxanthin on the expression of cell adhesion molecules on HUVECs

The effect of Fx on the expression of CAMs (including ICAM-1, VCAM-1, and E-selcetin) on HUVECs was analyzed byflow cytometry (Lu et al., 2014; Lian et al., 2016). HUVECs were seeded at a density of 1 × 10^5^ cells per well in a six-well plate for 24 h. Then the HUVECs were pretreated with various concentrations of Fx (0 μM, 1 μM, 5 μM, 10 μM, and 25 μM) for 24 h, followed by the addition of TNF-α (10 ng/ml) to stimulate the HUVECs for 4 h. HUVECs were collected and stained with mouse anti-human CD106 (PE-labeled), mouse anti-human CD54 (APC-labeled) and mouse anti-human CD62E (APC-labeled) antibody, respectively. Background staining was determined with an isotype-matched control antibody. Expression of E-selectin, ICAM-1, and VCAM-1 on HUVECs surface was detected by flow cytometry and analyzed by FlowJo software.

### HUVECs’ capillary tube formation assay

Matrigel was melted on the ice, followed by coating a 24-well plate (50 μl/well) and then polymerizing at 37°C for over 30 min. HUVECs (1 × 10^4^ cells) were suspended in 100 μl of the ECM medium with different concentrations of Fx (0 μM, 5 μM, 10 μM, and 25 μM), and were then seeded onto the 24-well plate pretreated with Matrigel and incubated for 6 h. Capillary-like structures were observed under an optical microscope. The tube formation and lengths, the number of loops, and branch nodes were quantified from three randomly selected fields per experiment.

### Effect of Fucoxanthin on NF-κB signaling pathway on HUVECs

To explore the effect of Fx on the TNF-α-stimulated NF-кB signaling pathway, HUVECs (1 × 10^5^ cells per well) were seeded in six-well plates for 24 h. The cells were pretreated with various concentrations of Fx (0 μM, 5 μM, 10 μM, 25 μM) for 24 h, followed by the addition of TNF-α (10 ng/ml) to stimulate the HUVECs for 4 h. After incubation, cells were lysed, and the protein expression levels of IкBα, p-IкBα (Ser 32), IKKα, IKKβ, p-IKKα/β (Ser 176/180), NF-кB p65, and p-NF-кB p65 (Ser 536) were analyzed by western blot. The nuclear translocation of NF-кB p65 was analyzed by immunofluorescence staining and photographed by confocal microscope. The western blot was described in Section 2.14.

### Effect of Fx on Epithelial-to-mesenchymal transition, PI3K/AKT, and FAK/Paxillin signaling pathways on cancer cells

To examine the effect of Fx on EMT and PI3K/AKT signaling pathways, MCF-7 cells (1 × 10^5^ cells per well) were seeded in 6-well plates. The cells were treated with a fresh medium containing various concentrations of Fx (0 μM, 5 μM, 10 μM, and 25 μM) for 24 h. After incubation, the protein expression levels of Zeb1, Snail1, Twist, N-cadherin, β-catenin, AKT1/2/3, p-AKT1/2/3 (AKT1-Tyr 315/AKT2-Tyr316/AKT3-Tyr312), PI3K, p-PI3K (Tyr 458), FAK, p-FAK (Tyr 397), p-PTK2 (Tyr 576/577), Paxillin, p-Paxillin (Tyr 118), CFL1 and p-CFL1 (Ser 3) were analyzed by western blot. The mRNA expression levels of SNAIL1, TWIST, ZEB1, FN1, VIM, and 18s rRNA were analyzed by quantitative reverse transcription-quantitative polymerase chain reaction (qRT-PCR), all primer sequences for qRT-PCR analysis are presented in Supplementary Table S1. The qRT-PCR was described in Section 2.15.

### Western blot analysis

Western blot analysis was performed as previously described (Lu et al., 2014; Lian et al., 2016; Cheng et al., 2018). Briefly, the cells were lysed with RIPA lysis buffer (Pierce) containing 1% phosphatase inhibitor and PMSF on the ice for 20 min. The lysates were centrifuged (18,000 *g* at 4°C for 15 min) to remove cell debris, and the supernatant was collected. The protein concentrations were measured by the BCA protein assay kit. The protein samples were denatured with loading buffer containing sodium dodecyl sulfate (SDS) at 100°C for 5 min. The equal amounts of denatured protein samples were separated on 6%–10% (w/v) sodium dodecyl sulfate polyacrylamide gel (SDS-PAGE), and then transferred to polyvinylidene fluoride (PVDF) membranes (Bio-Rad). The membranes were blocked in 5% BSA at room temperature for 1 h and then incubated with the primary antibodies (1:2,000 dilution) overnight at 4°C. The membranes were washed with TBST and then incubated with horseradish peroxidase (HRP)-linked secondary antibodies (1:10,000 dilution) at room temperature for 1 h. After being washed with TBST, the membranes were exposed to the ChemiDoc XRS System (Bio-Rad) to detect the expressions of the target proteins, which were enhanced by using the ECL Kit (Pierce) and quantified with Image Lab software (Bio-Rad), with normalization to GAPDH levels.

### RNA extraction and qRT-PCR assay

Total RNA was extracted using TRIzol reagent and resuspended in RNase-free water. The RNA concentrations were measured by using the BioDrop (BioDrop, United Kingdom), and mRNA was reverse transcribed into cDNA by using the TransScript^R^ All-in-One First-Strand cDNA Synthesis SuperMix (Transgen). The quantitative PCR (qPCR) was determined by using the PerfectStart^™^ Green qPCR SuperMix (Transgen), and carried out using the CFX96 touch real-time PCR detection system (iQ5, Bio-Rad) with the following thermocycling conditions: initial denaturation at 95°C for 30 sec; followed by 40 cycles of 95°C (5 s) and 60°C (30 s) (Cheng et al., 2018). The 18s rRNA was used as normalization control and the primer sequences for qPCR were presented in Supplementary Table S1.

### Animals and ethics statement

Specific pathogen-free female BALB/c mice (20 g–22 g, 6 weeks–8 weeks old) were purchased from the Shanghai SLAC Laboratory Animal Co., Ltd., (Lu et al., 2019a). Mice were housed in clean, pathogen-free rooms and had free access to pellet food and water. All animals used for studies were conducted in accordance with the Guide for the Care and Use of Laboratory Animals (National Research Council, 1996). The Laboratory Animal Ethics Committee of Minjiang University reviewed and approved all animal use procedures.

### *In vivo* pulmonary metastasis assay

The chemoprevention effect of Fx on 4T1 breast cancer BALB/c mice metastasis was examined. Experimental metastasis was performed as previously described (Lu et al., 2019a; Zhong et al., 2020). Briefly, 4T1 cells were harvested with the 0.05% trypsin-EDTA and washed and resuspended with PBS. The cell density was adjusted to 2.5 × 10^5^ cells per ml, and cell viability was greater than 95%, as determined by an automated cell counter (TC20, Bio-Rad). Then, female BALB/c mice were inoculated with 4T1 cells (5 × 10^4^ cells per mouse) *via* tail vein injection. Mice were pre-treated with Fx for 3 days [0 mg/kg, 1 mg/kg, 5 mg/kg, Fx dissolved in ethanol/soybean oil (1:9 v/v) solution, the volume of Fx is 100 μl per mouse, i.p. (intraperitoneal injection), q.d. (quaque die)] prior to inoculation with 4T1 cells, followed by continued treatment for 21 days. After the treatment, the mice were sacrificed. The lungs were removed and fixed in Bouin’s solution, and the number of colonies on the lung surface was counted. The lung, heart, liver, spleen, and kidney tissues of mice were formalin-fixed, paraffin-embedded, and stained with hematoxylin and eosin (H&E) for histological examination. Immunohistochemistry of paraffin-embedded lung tissues using antibodies to either PI3K, p-PI3K, AKT, or p-AKT. Additionally, blood samples were collected for routine blood tests. The percentage of cytotoxic T lymphocytes (Tc cells) and natural killer cells (NK cells) in peripheral blood and spleen were examined by flow cytometry.

### Statistical analysis

Data are described as mean ± standard deviation (SD). The significance of differences in experimental data was examined by analysis of variance (ANOVA) using the SPSS statistical software package, and the ANOVA was corrected for multiple comparisons by Dunnett’s Method. Statistical significance was calculated using biological replicates for all analyzed experimental data. In all statistical analyses, *p* < 0.05 was considered statistically significant and *p* < 0.01 was considered extremely statistically significant.

## Results

### Cytotoxicity of Fx

**FIGURE 1:**
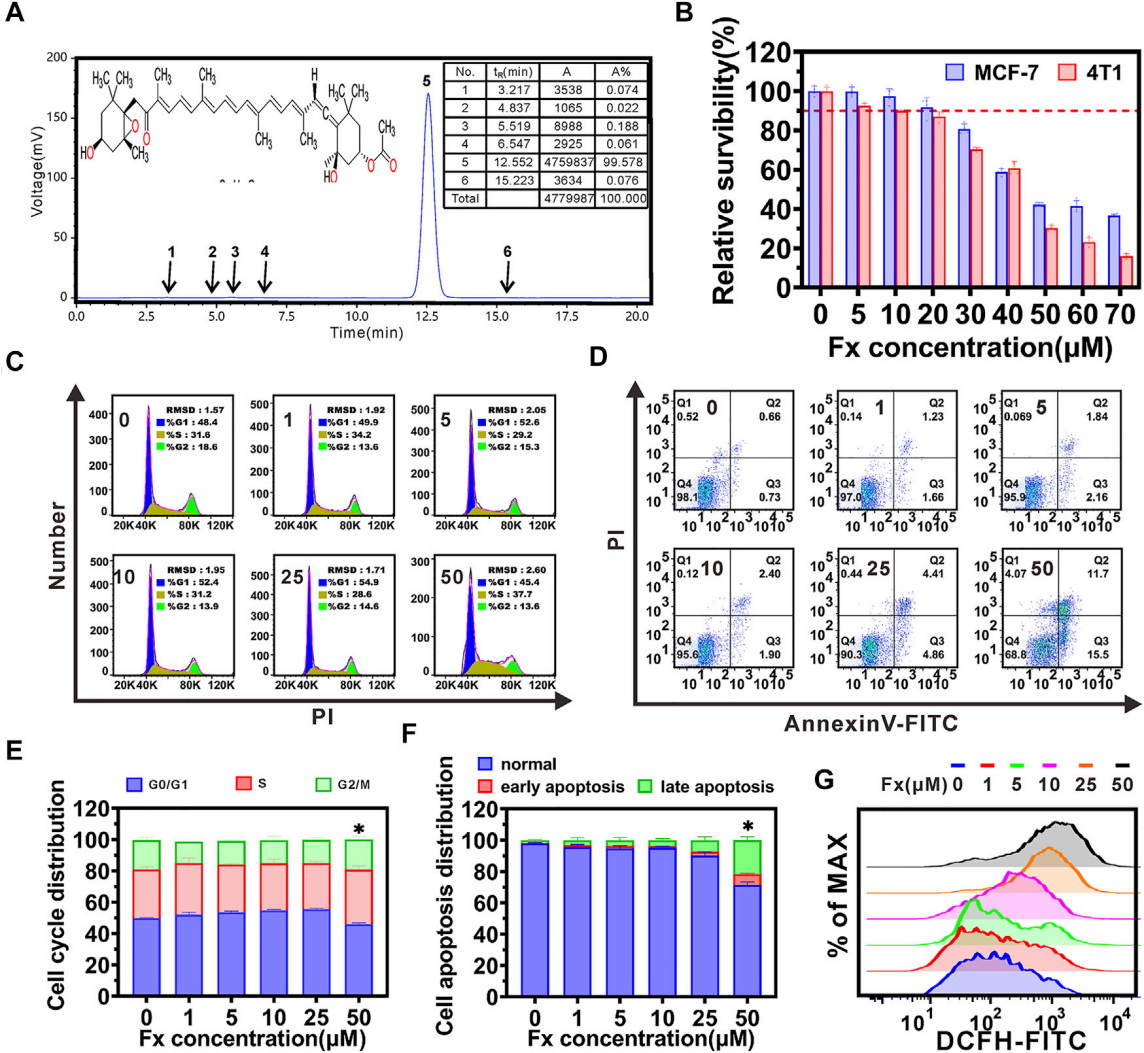
Inhibitory effects of Fucoxanthin (Fx). **(A)** Chemical structure and HPLC chromatogram of Fx. Peak at 12.5 min (peak 5) integration of the HPLC chromatogram indicated that the Fx is more than 99% purity. **(B)** The cytotoxicity of Fx to MCF-7 and 4T1 cells were determined by CCK8 assay, and relative survibility was expressed as percentage of control. **(C**,**D)** MCF-7 cells were incubated with Fx for 24 h, cell cycle distribution **(C)** and apoptosis **(D)** were analyzed by flow cytometry. **(E**,**F)** The distribution of cell cycle and apoptosis were shown as a histogram. **(G)** Intracellular ROS level in MCF-7 cells were determined by flow cytometry. Data are presented as mean ± SD (*n* = 3–6); * indicate *p* < 0.05 *vs*. Control.

Fx was provided by *Chengdu DeSiTe Biological Technology Co*., Ltd., (Chengdu, China). HPLC chromatogram showed that the purity of Fx was more than 99% (Figure 1A). The cytotoxicity of Fx was examined in MCF-7 and 4T1 cells, respectively. We found that Fx decreased cell viability in a concentration dependent manner, and its IC50 and IC10 (The concentration of drugs needed for 10% cell growth inhibition was defined as the IC10 value.) were greater than 40 μM and 20 μM, respectively (Figure 1B). Cell cycle and cell apoptosis analyses further supported that Fx, even at a concentration of 25 μM, did not significantly inhibit cell cycle distribution and induce apoptosis in MCF-7 and 4T1 cells (Figures 1C–F and Supplementary Figure S1). Only when the concentration reached 50 μM, Fx significantly induce apoptosis of MCF-7 cells by 28.7% ± 1.8%. Intracellular ROS level is involved in cell apoptosis, we found that a significant increase in ROS of MCF-7 was observed only when Fx concentration reached 25 μM (Figure 1G and Supplementary Figure S2), which was consistent with the results of cell cycle and apoptosis. These results indicated that Fx had weak cytotoxicity at concentrations less than 25 μM. Therefore, to demonstrate that the mechanism of cancer metastasis preventive effect of Fx is through inhibition of CTCs adhesion and transendothelial migration, instead of killing CTCs by cytotoxicity, we will choose a weakly cytotoxicity concentration of Fx for cancer metastasis prevention studies.

### Fx interrupts MCF-7 cells adhesion to HUVECs by inhibiting inflammatory factor-induced CAMs expression on endothelial cells

**FIGURE 2:**
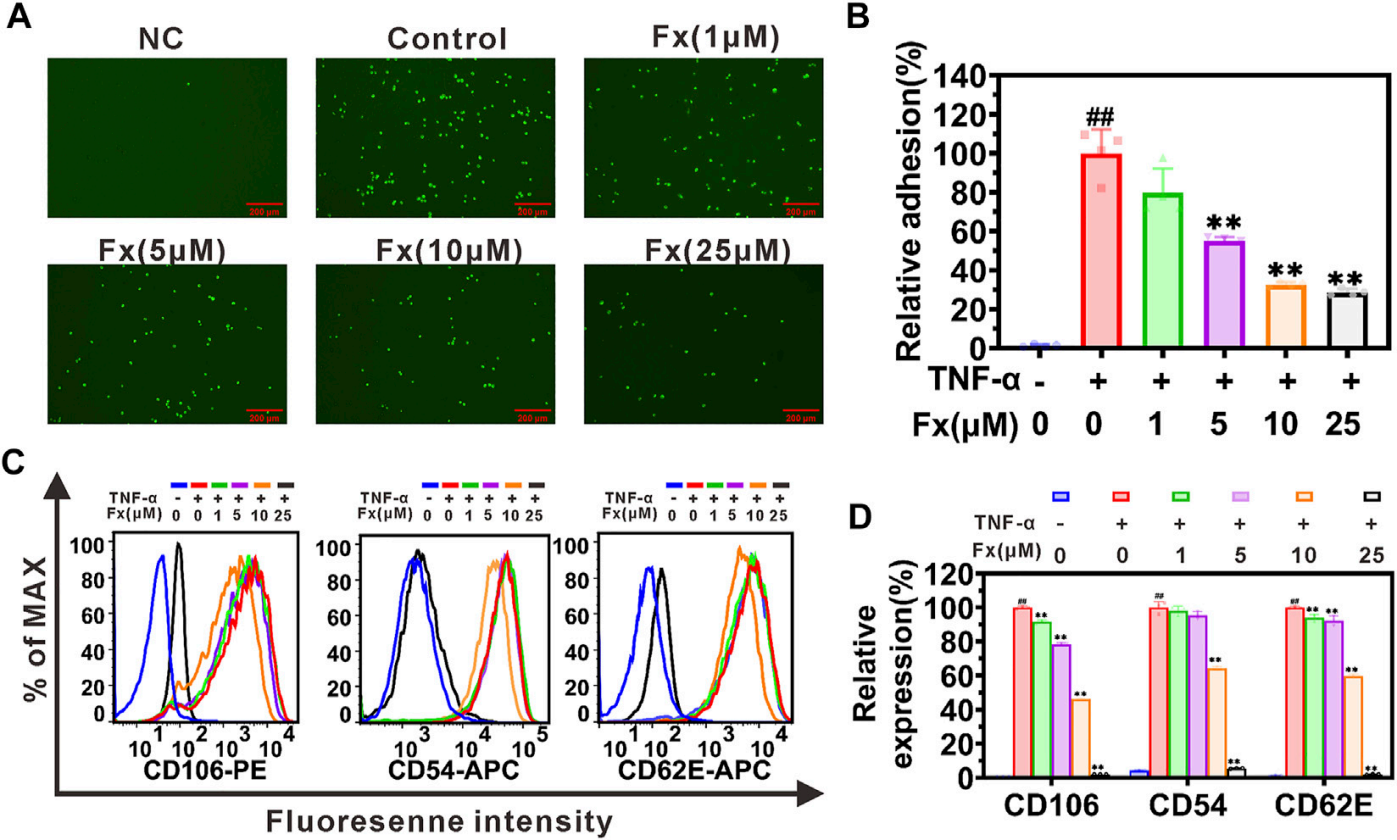
Fx interrupts MCF-7 cells adhesion to HUVECs by suppressing CAMs expression. **(A)** MCF-7 cells (green, labeled with Rhodamine 123) adhered to the HUVECs were photographed using a fluorescence microscope. **(B)** The relative adhesion of MCF-7 cells to HUVECs was expressed as percentage of control (TNF-α only). **(C)** HUVECs were pretreated with Fx for 24 h, followed by incubation with TNF-α (10 ng/ml) for another 4 h. Expression of CD106 (VCAM-1), CD54 (ICAM-1) and CD62E (E-selectin) on HUVECs were analyzed by flow cytometry. **(D)** The relative expression of CD106, CD54 and CD62E was expressed as percentage of the mean fluorescence intensity of control (TNF-α only). NC, negative control. Data are presented as mean ± SD (*n* = 3–6); ** indicate *p* < 0.01 *vs*. Control (TNF-α only); ^##^ indicate *p* < 0.01 *vs*. Negative control (TNF-α free).

The adhesion of CTCs to endothelial cells is an important initial step of distant cancer metastasis (Wan et al., 2015; Lu et al., 2019a). Here, we investigated the effect of Fx on the inflammatory factor TNF-α-induced heterogeneous adhesion of cancer cells MCF-7 to HUVECs. The adhesion of MCF-7 cells to HUVECs was quantified by fluorescence-labeled cell counts, and the results showed that Fx significantly inhibited adhesion in a concentration-dependent manner. Compared with the control (induced by TNF-α), the adhesion between MCF-7 and HUVECs was 79.8% ± 12.8%, 55.2% ± 2.0%, 32.6% ± 1.4%, and 28.7% ± 1.8%, respectively, with 1 μM, 5 μM, 10 μM and 25 μM of Fx (Figures 2A,B). The present study indicated that Fx significantly inhibited TNF-α-induced adhesion of MCF-7 to HUVECs at weakly cytotoxic concentrations.

Inflammatory factor induces the expression of CAMs on endothelial cells and plays an essential role in recruiting CTCs to adhere to the endothelial cells (Honn and Tang, 1992; Lu et al., 2019a). The effects of Fx on the expression of E-selectin (CD62E), ICAM-1 (CD54), and VCAM-1 (CD106) on HUVECs were determined by flow cytometry. As shown in Figures 2C,D, Fx significantly inhibited the expression of VCAM-1, ICAM-1, and E-selectin on HUVECs in a dose-dependent manner. This result prompted us to explore in more detail the molecular mechanism by which Fx interrupts the adhesion of MCF-7 cells to endothelial cells in the following studies.

### Fx inhibits CAMs expression on HUVECs by regulating the NF-кB signaling pathway

**FIGURE 3:**
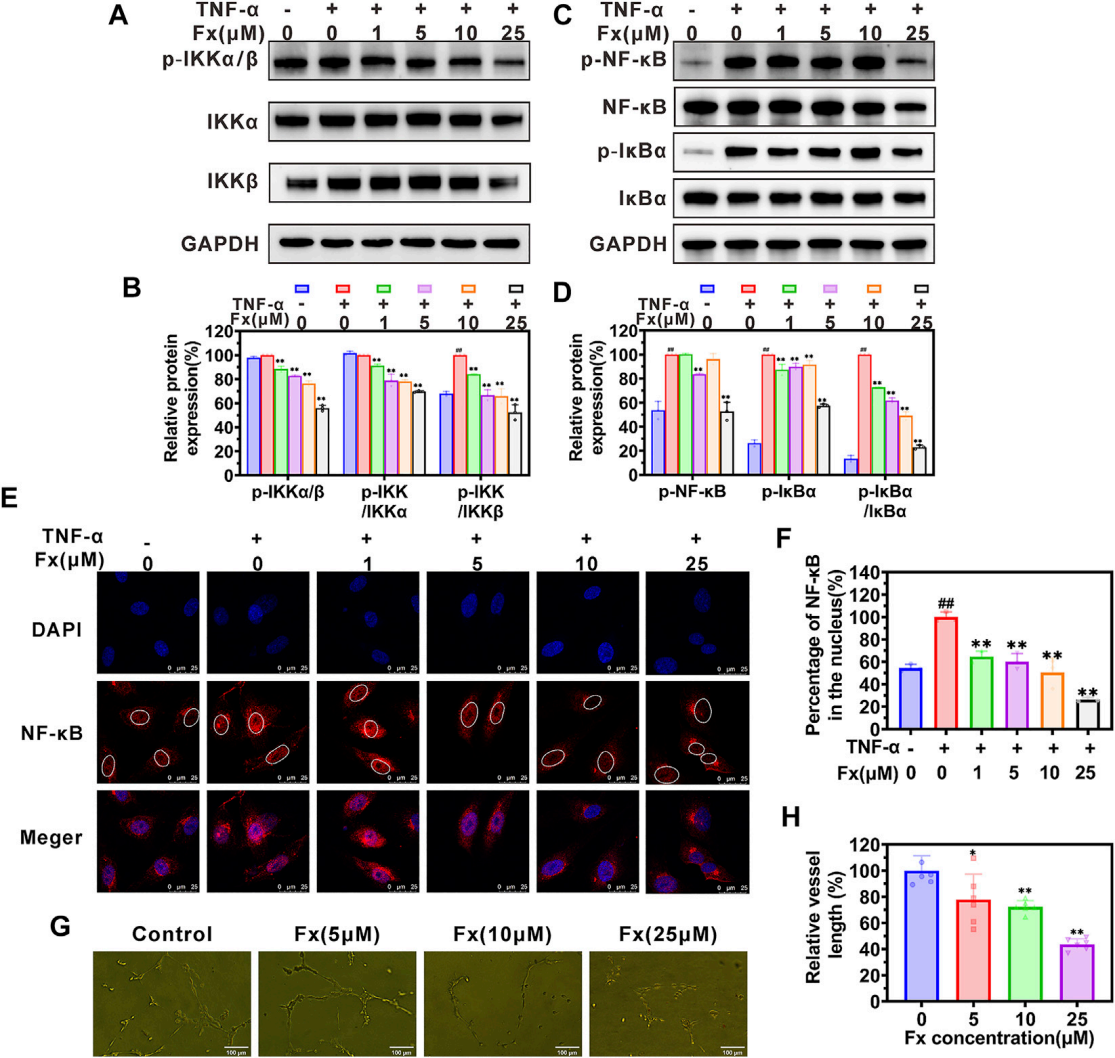
Fx inhibits the NF-κB pathway and capillary tube formation in HUVECs. **(A**–**D)** HUVECs were pretreated with Fx (0 μM, 1 μM, 5 μM, 10 μM and 25 μM) for 24 h and then stimulated with the TNF-α (10 ng/ml) for another 4 h. The expression levels of IкBα, p-IкBα (Ser 32), IKKα, IKKβ, p-IKKα/β (Ser 176/180), NF-кB p65 and p-NF-кB p65 (Ser 536) were analyzed by western blot, GAPDH was used as an internal control. Band intensity was quantified using Image Lab software and expressed as a percentage of control (TNF-α only). **(E)** The effect of Fx on the nuclear translocation of NF-кB p65 was analyzed by immunofluorescence staining and photographed by confocal microscope. Red represents NF-кB p65 (Alexa Fluor 594-labeled), and blue represent cell nucleus (DAPI-labeled). **(F)** The ratio of nuclear/total fluorescence intensity of NF-кB p65 (Alexa Fluor 594-labeled) was quantified using Fiji Image J software and expressed as a percentage of control (TNF-α only). Data are presented as mean ± SD (*n* = 3–6); ** indicate *p* < 0.01 *vs*. Control (TNF-α only); ^##^ indicate *p* < 0.01 *vs*. Negative control (TNF-α free). **(G)** Morphology assay of capillary tube formation in HUVECs treated with indicated concentrations of Fx for 6 h. **(H)** Vessel length was expressed as a percentage of control (Fx free). Data are presented as mean ± SD (*n* = 6); * indicate *p* < 0.05, ** indicate *p* < 0.01 *vs*. Control (Fx free).

The nuclear factor κB (NF-κB) signaling pathway plays a critical role in regulating the expression of CAMs (Read et al., 1997; Hoesel and Schmid, 2013; Lian et al., 2016). Here, we used a western blot to examine whether Fx inhibited TNF-α-induced CAMs expression on HUVECs by regulating the NF-кB signaling pathway. As shown in Figures 3A–D, TNF-α activates the NF-кB signaling pathway by promoting the phosphorylation of p-IкBα (Ser 32), p-IKKα/β (Ser 176/180), and p-NF-кB p65 (Ser 536), which was consistent with our previous report (Lian et al., 2016). When HUVECs were treated with Fx, although the ratio of p-NF-кB p65/NF-кB p65 was not significantly reduced (Supplementary Figure S3), it caused a significant concentration-dependent decrease in the total protein level of NF-кB p65 (Supplementary Figure S3) and phosphorylation levels of p-IкBα (Ser 32), p-IKKα/β (Ser 176/180) and p-NF-кB p65 (Ser 536) (Figures 3A–D), compared to the TNF-α stimulated HUVECs. We further analyzed the effect of Fx on NF-кB p65 nuclear translocation by immunofluorescence staining. Surprisingly, Fx significantly inhibited the nuclear translocation of NF-кB p65 in HUVECs in a dose-dependent manner (Figures 3E,F), suggesting that Fx inhibits NF-κB signaling pathway and induces NF-кB p65 degradation by blocking NF-кB p65 nuclear translocation. These results indicated that Fx down-regulating ICAM-1, VCAM-1, and E-selectin expression on HUVECs is closely associated with the NF-κB signaling pathway.

Cancer angiogenesis and growth are required for cancer growth and metastasis (Huinen et al., 2021). We further evaluated the effect of Fx on capillary tube formation. As shown in Figure 3G, an obvious capillary tube morphology was observed in the control group. After co-incubation with Fx, the morphology of the capillary tube was destroyed, and the vessel length was obviously reduced (Figure 3H). The result further indicated that Fx has a potential inhibitory effect on angiogenesis.

### Fx suppresses migration, invasion, and transendothelial migration of MCF-7 cells

**FIGURE 4:**
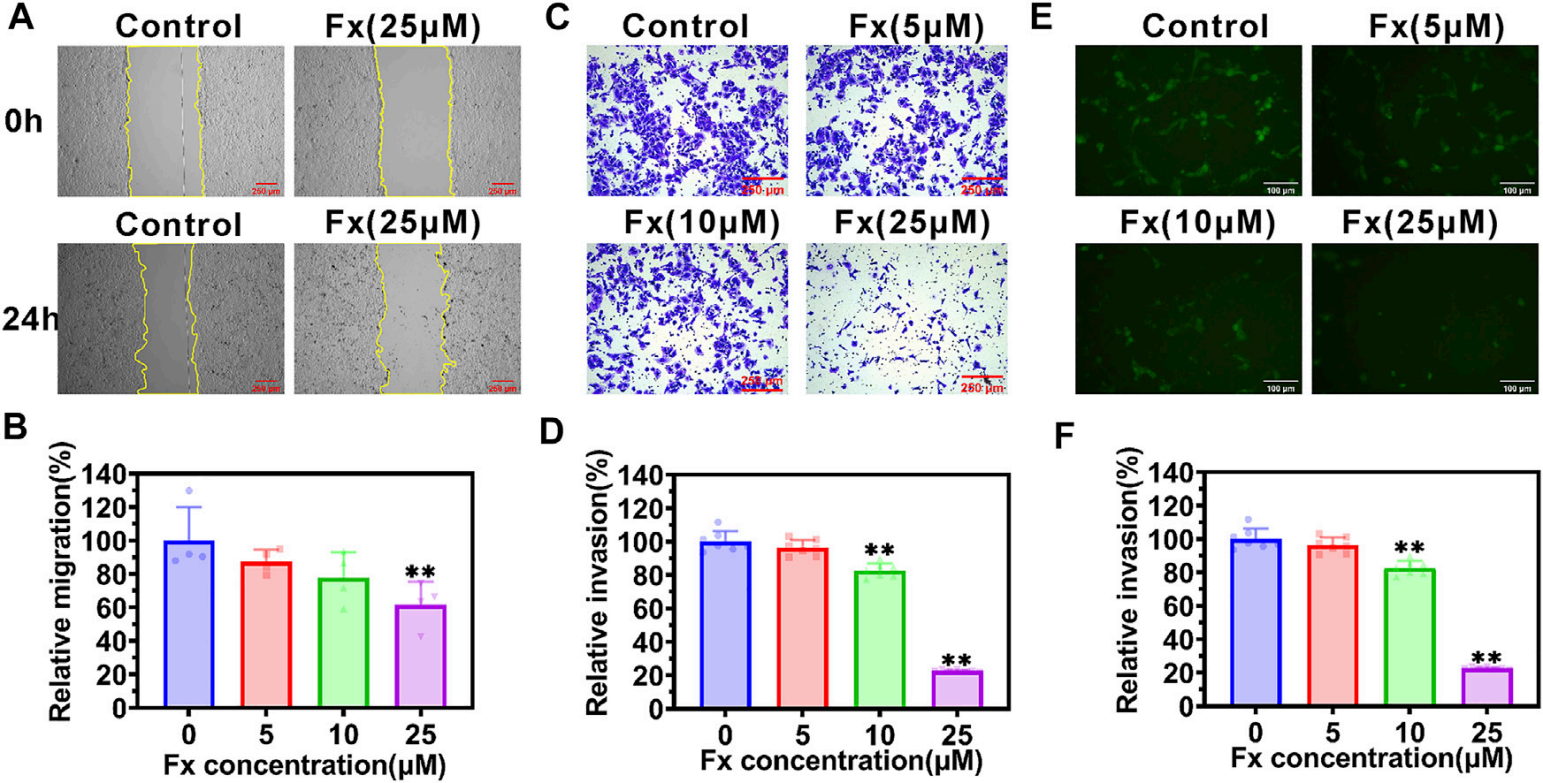
Fx inhibits migration, invasion, and transendothelial migration of MCF-7 cells. **(A**,**B)** Cell migration ability was determined by a wound-healing assay in MCF-7 cells treated with indicated concentrations of Fx for 24 h. The relative migration area was calculated as a percentage of control (Fx free). The yellow solid lines indicate the areas of migration. **(C**,**D)** Cell invasion ability was determined by transwell assay in MCF-7 cells treated with indicated concentrations of Fx for 24 h. The relative invasion rate was calculated as a percentage of control (Fx free). **(E**,**F)** Transendothelial migration analysis showed that Fx significantly inhibited the GFP-labeled MCF-7 cells (green) across the HUVECs. Data are presented as mean ± SD (*n* = 3–6); ** indicate *p* < 0.01 *vs*. Control (Fx free).

The traversing of CTCs across the vascular barrier is another crucial step in the metastasis process, and the aggressiveness of cancer cells plays an important role in this step (Labelle and Hynes, 2012; Reymond et al., 2013; Harney et al., 2015). We examined the effect of Fx on aggressive behaviors of MCF-7 cells *in vitro*. The wound-healing assay showed that Fx significantly inhibited the MCF-7 cell migration in a concentration-dependent manner (Figures 4A,B). Moreover, the Transwell assay showed that Fx markedly inhibited the MCF-7 cell invasion in a concentration-dependent manner, especially in the 25 μM treatment group, in which the invasion rate was only 22.2%, compared with the control group (Figures 4C,D). To further evaluation of the effect of Fx on the transendothelial migration of MCF-7 cells, we seeded HUVECs into Transwell upper chambers coated with Matrigel and allowed them to grow to confluence. As a result, Fx at 5 μM, 10 μM, and 25 μM caused a significant concentration-dependent decrease in GFP-labeled MCF-7 cells transendothelial migration rate by 89.5% ± 10.6% (5 μM), 64.2% ± 9.3% (10 μM) and 20.9% ± 3.3% (25 μM), respectively, compared with the control group (Figures 4E,F). These results showed that Fx significantly inhibited invasion and transendothelial migration of MCF-7 at weakly cytotoxic concentrations.

### Fx suppresses cancer cell metastasis by inhibiting EMT, PI3K/AKT, and FAK/Paxillin signaling pathways

**FIGURE 5:**
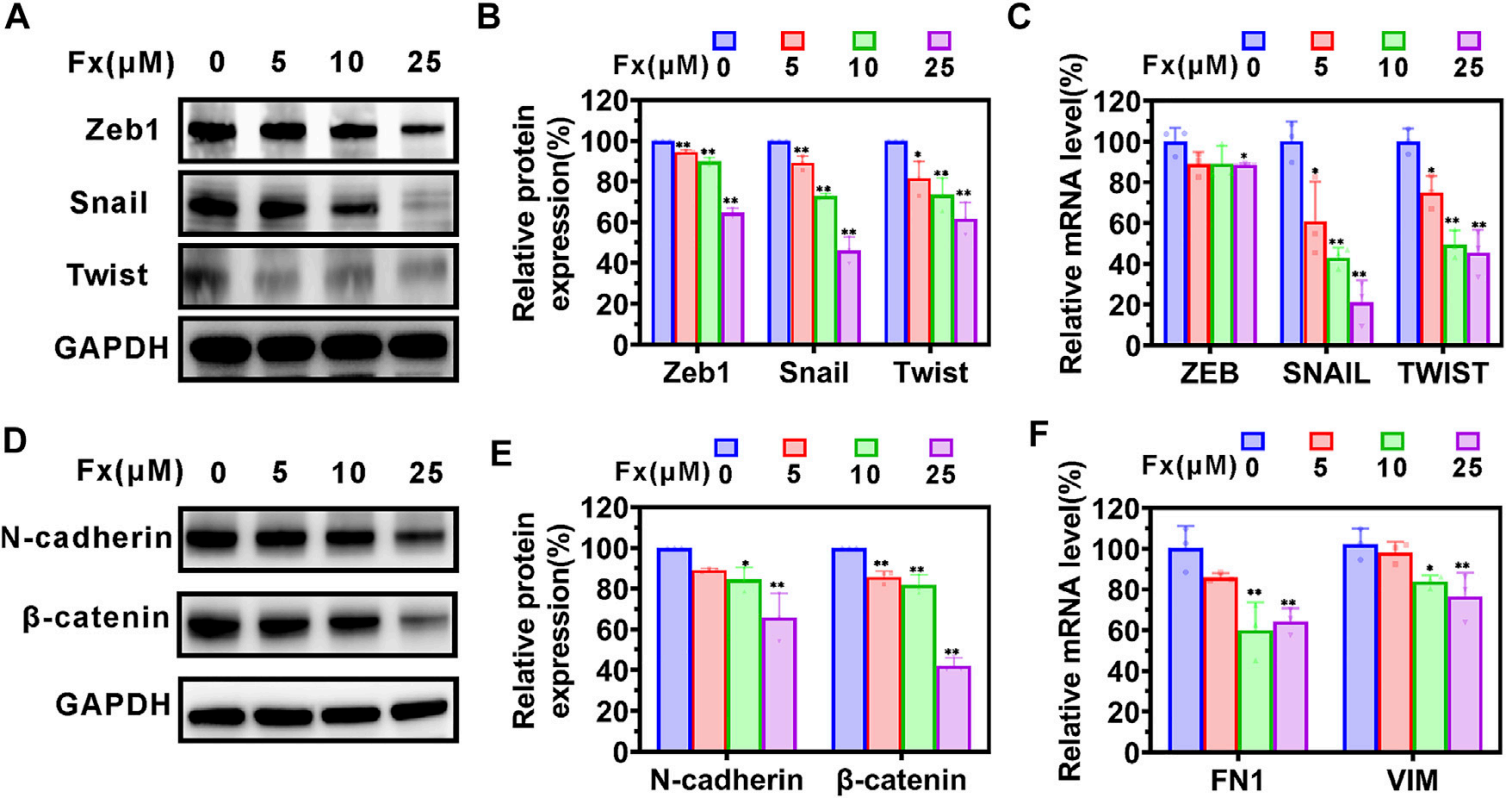
The effects of Fx on EMT-related protein in MCF-7 cells. MCF-7 cells were treated with Fx for 24 h, and the expression levels of EMT-related protein and mRNA were analyzed by western blot and qRT-PCR, respectively. **(A**–**C)** Western blot and qRT-PCR analysis showed that Fx significantly inhibited the expression of EMT transcription factors ZEB, SNAIL, and Twist. **(D**–**F)** Western blot and qRT-PCR analysis showed that Fx significantly inhibited the expression of mesenchymal markers N-cadherin, β-catenin, FN1, and VIM. FN1, fibronectin; VIM, vimentin. Data are presented as mean ± SD (*n* = 3); * indicate *p* < 0.05, ** indicate *p* < 0.01 *vs*. Control (Fx free).

EMT plays an important role in enhancing the metastatic capacity of cancer cells, during which cancer cells acquire mesenchymal invasive properties and lose their epithelial characteristics (Thiery et al., 2009; Cheng et al., 2018). We examined the effects of Fx on the expression of EMT-related protein and mRNA on MCF-7 cells using western blot and qRT-PCR, respectively. The results are shown in Figure 5, Fx caused a concentration-dependent inhibition of Zeb1, Snail, Twist, N-cadherin, and β-catenin proteins expression, compared with the control group. Similarly, the mRNA transcription levels of ZEB1, SNAIL1, TWIST, FN1, and VIM were reduced by Fx treatment (Figure 5).

**FIGURE 6:**
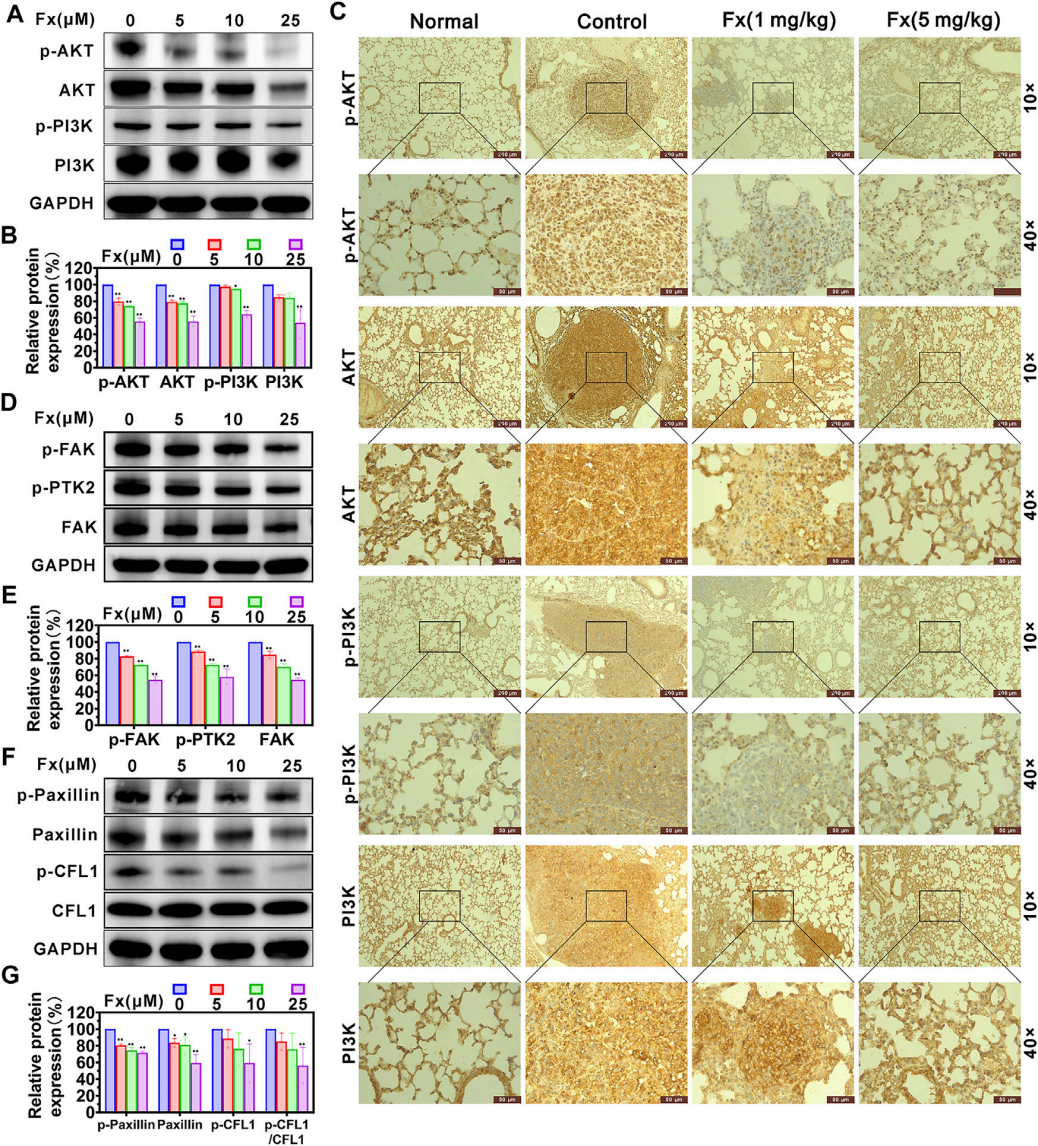
Fx inhibits PI3K/AKT and FAK/Paxillin signaling pathways in MCF-7 cells and lung metastases. MCF-7 cells were treated with Fx for 24 h, and the expression levels of AKT1/2/3, p-AKT1/2/3 (AKT1-Tyr 315/AKT2-Tyr316/AKT3-Tyr312), PI3K, p-PI3K (Tyr 458), FAK, p-FAK (Tyr 397), p-PTK2 (Tyr 576/577), Paxillin, p-Paxillin (Tyr 118), CFL1 and p-CFL1 (Ser 3) were analyzed by western blot, GAPDH was used as an internal control. Band intensity was quantified using Image Lab software and expressed as a percentage of control (Fx free). **(A**,**B)** Fx significantly inhibited the total and phosphorylation levels of PI3K and AKT proteins in MCF-7 cells in dose-dependent manners. **(C)** BALB/c mice were pre-treated with Fx for 3 days (0 mg/kg, 1 mg/kg, 5 mg/kg, the volume of Fx is 100 μl per mouse, i.p., q.d.) prior to inoculation with 4T1 cells (5 × 104 cells/mouse, tail vein injection), followed by continued treatment for 21 days. Representative immunohistochemical staining for AKT1/2/3, p-AKT1/2/3 (AKT1-Tyr 315/AKT2-Tyr316/AKT3-Tyr312), PI3K, p-PI3K (Tyr 458) in lung metastases are shown. **(D**–**G)** Fx significantly inhibited the total and phosphorylation levels of FAK and Paxillin proteins in MCF-7 cells in dose-dependent manners. Data are presented as mean ± SD (*n* = 3); * indicate *p* < 0.05, ** indicate *p* < 0.01 *vs*. Control (Fx free).

A series of studies have demonstrated that PI3K/AKT signaling pathway is actively involved in the migration process of metastatic cancer cells, including the regulation of cytoskeleton-remodeling proteins (such as Vimentin and F-actin) and EMT–activating proteins (such as Snail and Twist) that specifically regulate cell motility (Xue and Hemmings, 2013; Lamouille et al., 2014). We examined the effects of Fx on the expression of PI3K/AKT signaling pathway and cytoskeleton-regulating proteins in MCF-7 cells using western blot. As shown in Figures 6A,B, Fx significantly inhibited PI3K, p-PI3K (Tyr 458), AKT1/2/3, and p-AKT1/2/3 (AKT1-Tyr 315/AKT2-Tyr316/AKT3-Tyr312) expression with a concentration-dependent manner, compared with the control group. However, the ratios of pAKT/AKT and p-PI3K/PI3K were not significantly different (Supplementary Figure S4). These results may suggest that Fx inhibits PI3K/AKT signaling pathway by inducing degradation of total protein. We further evaluated the effect of Fx for the PI3K/AKT pathway in the lungs of mice based on immunohistochemical staining. Interestingly, PI3K, p-PI3K (Tyr 458), AKT1/2/3, and p-AKT1/2/3 were highly expressed in lung tumor metastases compared with healthy lung tissues, but their expression was significantly decreased in lung tumor metastases after Fx treatment (Figure 6C and Supplementary Figure S5), suggesting that Fx also inhibited PI3K/AKT signaling pathway in cancer cells *in vivo*. Furthermore, the FAK/Paxillin signaling axis also plays an important role in promoting cancer cell migration and invasion (Lietha et al., 2007; Du et al., 2014). FAK regulates the function of Paxillin through phosphorylation, and hosphor-Paxillin serves as a critical docking site for recruiting other signaling molecules to focal adhesions, promoting cell motility, invasion, and survival (Yu et al., 2015). In present study, we found that Fx significantly down-regulation of FAK, p-FAK (Tyr 397), p-PTK2 (Tyr 576/577), Paxillin, p-Paxillin (Tyr 118) and p-CFL1 (Ser 3) expression with a concentration dependent manner (Figures 6D–G), but the ratios of p-FAK/FAK, p-PTK2/FAK and p-Paxillin/Paxillin were not significantly different (Supplementary Figure S6). Dephosphorylated cofilin cannot be transported from the nucleus to the cytoplasm, so it cannot promote the regeneration of actin filaments by severing preexisting filaments (Nebl et al., 1996; Condeelis, 2001). Thus, these data combined indicated that Fx inhibits cancer cells invasion and transendothelial migration by regulating EMT, PI3K/AKT and FAK/Paxillin signaling pathways.

### Fx inhibits 4T1 breast cancer pulmonary metastasis

**FIGURE 7:**
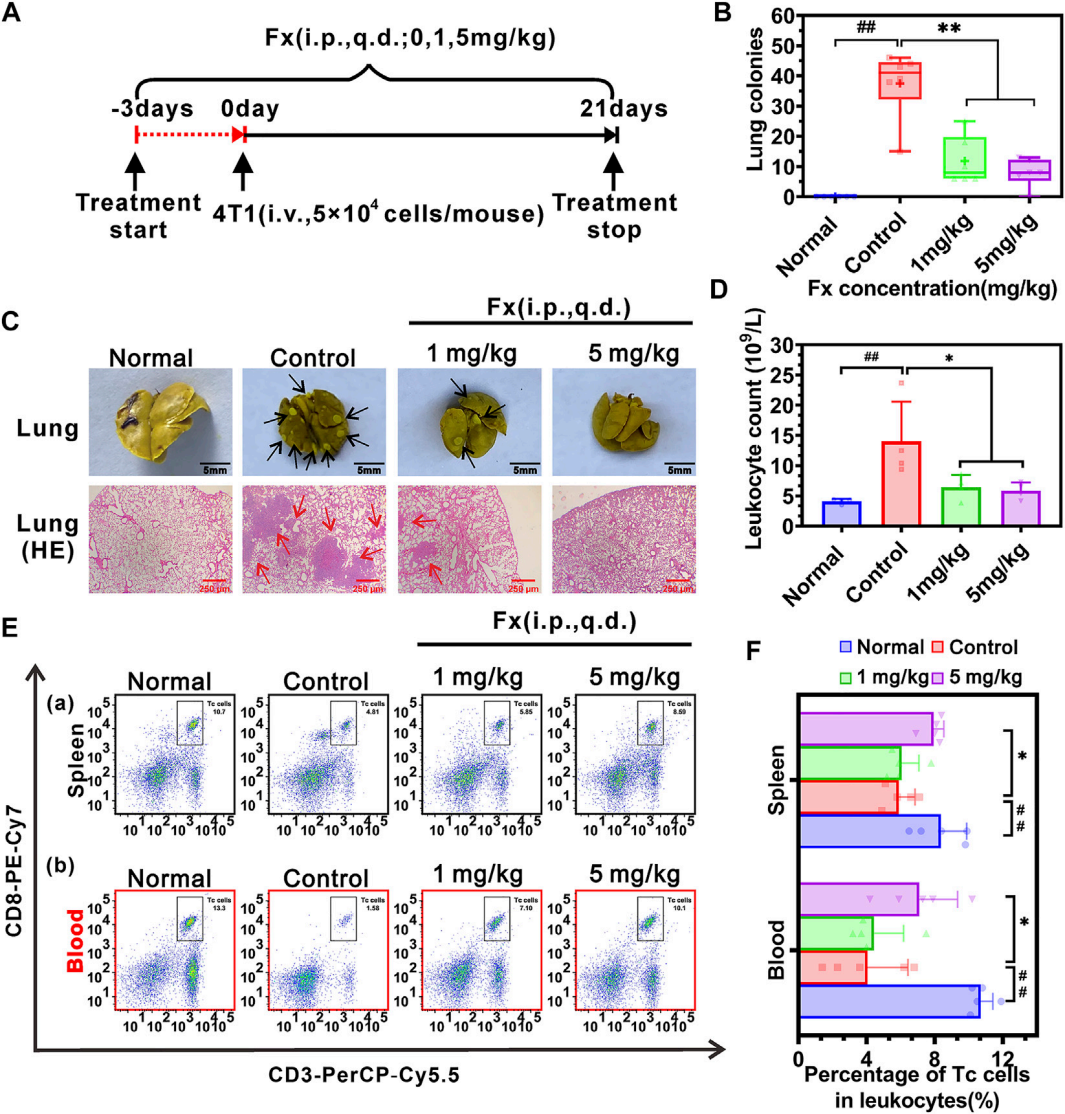
Effects of Fx *in vivo* breast cancer pulmonary metastasis in immunocompetent BALB/c mice. **(A)** Schematic diagram of the experimental protocol of Fx for treatment of cancer metastasis *in vivo*. Mice were pre-treated with Fx for 3 days (0 mg/kg, 1 mg/kg, 5 mg/kg, the volume of Fx is 100 μl per mouse, i.p., q.d.) prior to inoculation with 4T1 cells (5 × 10^4^ cells/mouse, tail vein injection), followed by continued treatment for 21 days. **(B)** Fx significantly decreased the number of pulmonary metastatic nodules in a dose-dependent manner. **(C)** Representative photos of the lungs showing surface metastasis and H&E staining in Fx-treated mice. The black arrows indicate the breast cancer pulmonary nodules, and the red arrows indicate the breast cancer metastasis area. **(D)** Routine blood tests showed that the Fx significantly reduced the number of leukocytes to the normal range. **(E)** The percentage of cytotoxic T lymphocytes (Tc cells, CD45^+^CD3^+^CD8^+^) in peripheral blood and spleen of mice was detected by flow cytometry. **(F)** Quantitative analysis showed that the Fx significantly increased the percentage of Tc cells in peripheral blood and spleen and approached the normal range. Data are presented as mean ± SD (*n* = 6); * indicate *p* < 0.05, ** indicate *p* < 0.01 *vs*. Control (Pulmonary metastatic mice); ^#^ indicate *p* < 0.05, ^##^ indicate *p* < 0.01 *vs*. Normal (Healthy mice).

To further examine the effect of Fx on preventing 4T1 cells-mediated pulmonary metastasis *in vivo*, female BALB/c mice were pretreated with Fx (0, 1, 5 mg/kg) for 3 days and inoculated with 4T1 cells *via* tail vein injection, and then continued treatment with Fx for 21 days (Figure 7A). As shown in Figure 7B, Fx at 1 mg/kg and 5 mg/kg significantly and reduces 4T1 cell-induced lung metastatic nodules. Moreover, Histopathological hematoxylin and eosin (H&E) staining showed that lung metastatic lesions in the Fx-treated groups were significantly reduced compared with the control group (Figure 7C). Importantly, 3 weeks of Fx treatment did not produce any observable side effects in the mice. As shown in Supplementary Figure S7, Fx treatment did not significantly alter the body weight of the mice (Supplementary Figure S7A), and H&E staining of the heart, liver, spleen, and kidney showed that the histological features of the Fx-treated mice were normal (Supplementary Figure S7B). The *in vivo* data indicated that Fx at weakly toxic doses shows a remarkable inhibitory effect on 4T1 breast cancer pulmonary metastasis.

Furthermore, improving immune system function is conducive to suppressing cancer metastasis *in vivo* (Lian et al., 2019a; Lian et al., 2019b). Routine blood tests on mice showed that the pulmonary metastatic mice (Control group) significantly increased the number of leukocytes in peripheral blood, compared with the healthy mice (Normal group). Fx treatment significantly reduced the number of leukocytes to the normal range (Figure 7D). To examine the effect of Fx on cell-mediated immunity, the percentage of cytotoxic T lymphocytes (Tc cells) and natural killer cells (NK cells) in peripheral blood and spleen were analyzed by flow cytometry. We found that the pulmonary metastatic mice (Control group) significantly reduced the percentage of Tc cells (CD45^+^CD3^+^CD8^+^ cells) in peripheral blood and spleen, compared with the healthy mice. However, Fx treatment significantly increased the percentage of Tc cells in peripheral blood and spleen and approached the normal range (Figures 7E, F). No significant differences were found in the percentage of NK cells (CD45^+^CD49b+ cells) in peripheral blood and spleen of the Fx-treated group compared with the control group (Supplementary Figures S7C,D). These results indicated that Fx suppresses cancer metastasis and enhances the anti-cancer immune response by unleashing Tc cells in the immune system.

## Discussion

**FIGURE 8:**
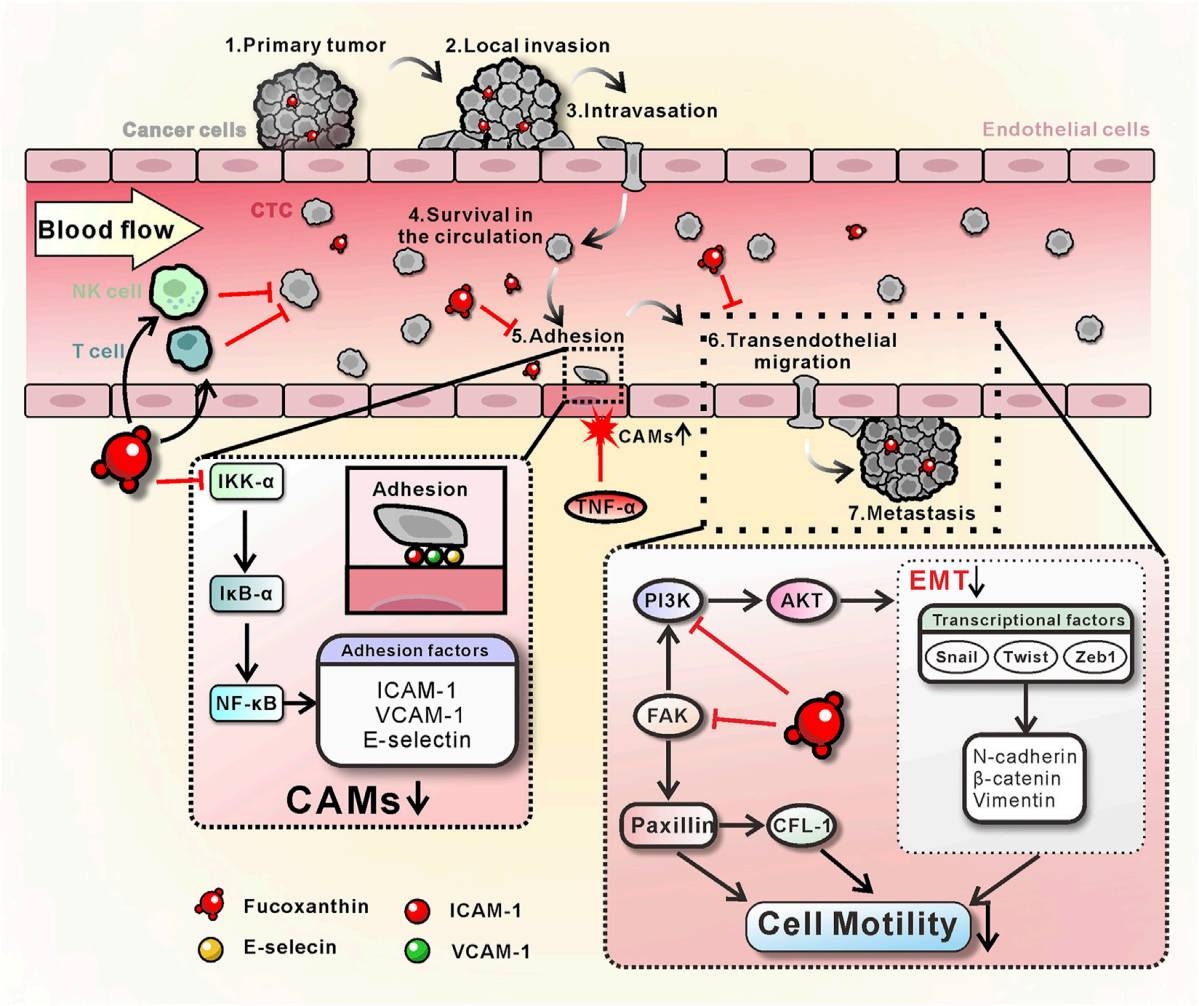
Schematic diagram of the mechanism of Fx prevents breast cancer metastasis. On the one hand, Fx inhibits TNF-α-induced CAMs (ICAM-1, VCAM-1, and E-selectin) expression on endothelial cells by inhibiting the NF-кB signaling pathway, resulting in the interruption of CTCs adhesion to the vascular endothelium. On the other hand, Fx reduces EMT and cancer cell motility by regulating PI3K/AKT and FAK/Paxillin signaling pathways, resulting in the inhibition of cancer cell transendothelial migration. Furthermore, Fx enhances antitumor immune responses by substantially increasing the subsets of Tc cells in the peripheral immune system. These beneficial effects of Fx collectively resulted in the suppression of CTCs-induced cancer metastasis.

The present studies demonstrated that the Fx has a significant preventive effect on cancer metastasis by interrupting CTCs adhesion and transendothelial migration. However, the same concentration of Fx did not significantly alter the cell viability, cell cycle, apoptosis, and ROS of MCF-7 cells, thus excluding the possibility that Fx inhibits CTCs adhesion and transendothelial migration through cytotoxicity. Further study showed evidence that the mechanism of Fx inhibits the expression of CAMs on endothelial cells by inhibiting the NF-кB signaling pathway by down-regulating the phosphorylation level of IKK-α/β, IкB-α, and NF-кB p65, and inhibits transendothelial migration of MCF-7 cells by regulating EMT, PI3K/AKT and FAK/Paxillin signaling pathways. We also showed that Fx enhances antitumor immune responses by substantially increasing the subsets of Tc cells in the peripheral immune system. These beneficial effects of Fx (Figure 8) collectively resulted in the inability of CTCs to adhesion to endothelium and transendothelial migration, effectively preventing cancer metastasis.

Inflammatory cytokines stimulate the expression of endothelial cell surface CAMs to bind to the corresponding ligands on CTCs, which can then promote the rolling and adhesion of CTCs on the vascular endothelium (Lauri et al., 1991; Labelle and Hynes, 2012). Previous research also found that ICAM-1 promotes CTCs adhesion to pulmonary microvessels and transendothelial migration in breast cancer pulmonary metastasis (Taftaf et al., 2021). It has now been demonstrated that NF-кB plays an essential role as a dimeric transcription factor in the inflammatory cytokine-induced expression of CAMs (including ICAM-1, VCAM-1, and E-selectin) (Hoesel and Schmid, 2013; Lian et al., 2016). In the resting state of the NF-κB pathway, the activity of NF-κB protein is inhibited by binding to IκB protein. In the presence of inflammatory cytokines (including IL-1β, TNF-α, and LPS, et al.), the IKK complex (IKKβ and IKKα) is activated and leads to phosphorylates IκB proteins. Phosphorylated IκB is degraded by ubiquitination, thereby releasing the NF-κB protein (Hayden and Ghosh, 2008). Free NF-κB protein is further activated by post-translational modifications and transported into the nucleus to induce target gene expression (Perkins, 2006). Since the gene promoters of ICAM-1, VCAM-1, and E-selectin contain at least one кB binding site, the activation of the NF-κB signaling pathway can promote their expression (Read et al., 1997; Lian et al., 2016). The present study clearly demonstrated that Fx interrupts CTCs adhesion to HUVECs by inhibiting the TNF-α-induced CAMs expressions on endothelial cells (Figure 2). Further studies showed that the mechanism of Fx decreases the expression of CAMs by inhibiting the NF-кB signaling pathway by down-regulating the phosphorylation level of IKK-α/β, IкB-α, and NF-кB p65, and the nuclear translocation of NF-кB p65 (Figure 3).

Transendothelial migration is a critical process in cancer metastasis. During transendothelial migration, CTCs first adhere to endothelial cells, open endothelial cell junctions, induce endothelial retraction, and are then inserted into the endothelial monolayer between endothelial cells, this process requires a tumor cell to acquire enhanced motility (Reymond et al., 2012). It is now widely accepted that EMT is closely related to the loss of epithelial characteristics and the acquisition of mesenchymal invasive properties, resulting in enhanced motility and invasive potential of cancer cells (Lamouille et al., 2014). The EMT process is very complex, mediated by transcription factors such as SNAIL, ZEB, and Twist, activating mesenchymal markers (including N-cadherin, β-catenin, Fibronectin, and Vimentin) that play an important role in enhancing cancer cell motility (Thiery and Sleeman, 2006; Mani et al., 2008). In the current study, Fx significantly inhibited the expressions of N-cadherin, β-catenin, Fibronectin, and Vimentin in cancer cells, possibly by downregulating the expression of transcription factors SNAIL, ZEB, and Twist (Figure 5).

The function of the PI3K/AKT signaling pathway in human physiology and disease has been intensively investigated. It has been found that inhibition the of PI3K/AKT signaling pathway reduces cancer cell motility and attenuates malignant progression of cancer metastasis (Xue and Hemmings, 2013). Furthermore, activation of the PI3K pathway promotes cancer cells to undergo EMT, and inhibition of AKT reduces the expression level of EMT-related protein SNAIL1 (Lamouille et al., 2014). TGFβ-induced EMT is prevented by pharmacological inhibition of PI3K, indicating that the PI3K/AKT signaling pathway plays an essential role in the EMT (Bakin et al., 2000; Julien et al., 2007; Lamouille et al., 2014). Therefore, targeting the PI3K/AKT pathway with small molecule inhibitors may be an important strategy for cancer metastasis chemoprevention. FAK regulates the function of Paxillin through phosphorylation and plays a critical role in cytoskeletal organization, focal adhesion formation, and cell motility (Lietha et al., 2007; Du et al., 2014; Yu et al., 2015). In the present study, we found that Fx downregulated the total and phosphorylation levels of PI3K, AKT, FAK, and Paxillin proteins in MCF-7 cells in dose-dependent manners (Figure 6). These data demonstrated that Fx inhibits PI3K/AKT and FAK/Paxillin signaling pathways by inducing degradation of total protein, and the mechanism is similar to that reported in previous studies (Shi et al., 2014; Zhao et al., 2017; Zhang et al., 2022). Taken together, these results strongly support that Fx inhibits invasion and transendothelial migration of CTCs by inhibiting EMT, PI3K/AKT, and FAK/Paxillin signaling pathways.

## Conclusion

In the present study, we demonstrated that the weakly cytotoxic Fx prevents breast cancer metastasis by interrupting CTCs adhesion to endothelium and transendothelial migration (Figure 8). Mechanistically, Fx interrupts CTCs adhesion to vascular endothelium by inhibiting TNF-α-induced CAMs expression on endothelial cells, which regulates the NF-кB signaling pathway by inhibiting the phosphorylation level of IKK-α/β, IкB-α, and NF-кB p65. Fx inhibits transendothelial migration of CTCs by inhibiting EMT, PI3K/AKT, and FAK/Paxillin signaling pathways. Moreover, Fx enhances antitumor immune responses by substantially increasing the subsets of Tc cells in the peripheral immune system. Unlike chemotherapeutic agents that kill cancer cells through extreme cytotoxicity while damaging the immune system and producing many intolerable side effects, Fx showed low cytotoxicity and comprehensive cancer metastatic preventive effects. This new finding provides a basis for the application of Fx in cancer metastatic chemoprevention and suggests that interruption of the CTCs adhesion to endothelium and transendothelial migration may serve as a new avenue for cancer metastatic chemoprevention.

## Data Availability

The original contributions presented in the study are included in the article/Supplementary Materials, further inquiries can be directed to the corresponding authors.
