# Supplementary material for: Fucoxanthin prevents breast cancer metastasis by interrupting circulating tumor cells adhesion and transendothelial migration
Source: Front Pharmacol. 2022 Sep 9;13:960375. doi: 10.3389/fphar.2022.960375 (PMC9500434; doi:10.3389/fphar.2022.960375)
Supplement: Supplementary file 1 [file DataSheet1.docx]

Supplementary Material

## Supplementary Tables

**Supplementary Table 1.** Primer sequences used for reverse transcription-quantitative polymerase chain reaction analyses of human colorectal cancer cells.

| **Gene** | **Primer sequence (5’-3’)** |
| --- | --- |
| SNAIL1 | F: GCTGCTACAAGGCCATGTCCGG |
|  | R: CTTGGTGCTTGTGGAGCAGGGAC |
| ZEB1 | F: ATCCTGGGGCCTGAAGCTCAGG |
|  | R: TGGTGTGCCCTGCCTCTGGT |
| FN1 | F: TGCAAGGCCTCAGACCGGGT |
|  | R: GCGCTCAGGCTTGTGGGTGT |
| VIM | F: TTCCAAGCCTGACCTCACGGCTG |
|  | R: TTCCGGTTGGCAGCCTCAGAGA |
| TWIST | F: TCCGCGTCCCACTAGCAGGC |
|  | R: CGCCCCACGCCCTGTTTCTT |
| 18s | F: AGAAACGGCTACCACATCCA |
|  | R: CCCTCCAATGGATCCTCGTT |

FN, Fibronectin; SNAIL, snail family transcriptional repressor; ZEB, Zinc-finger E-box-binding homeobox; VIM, Vimentin.

## Supplementary Figures


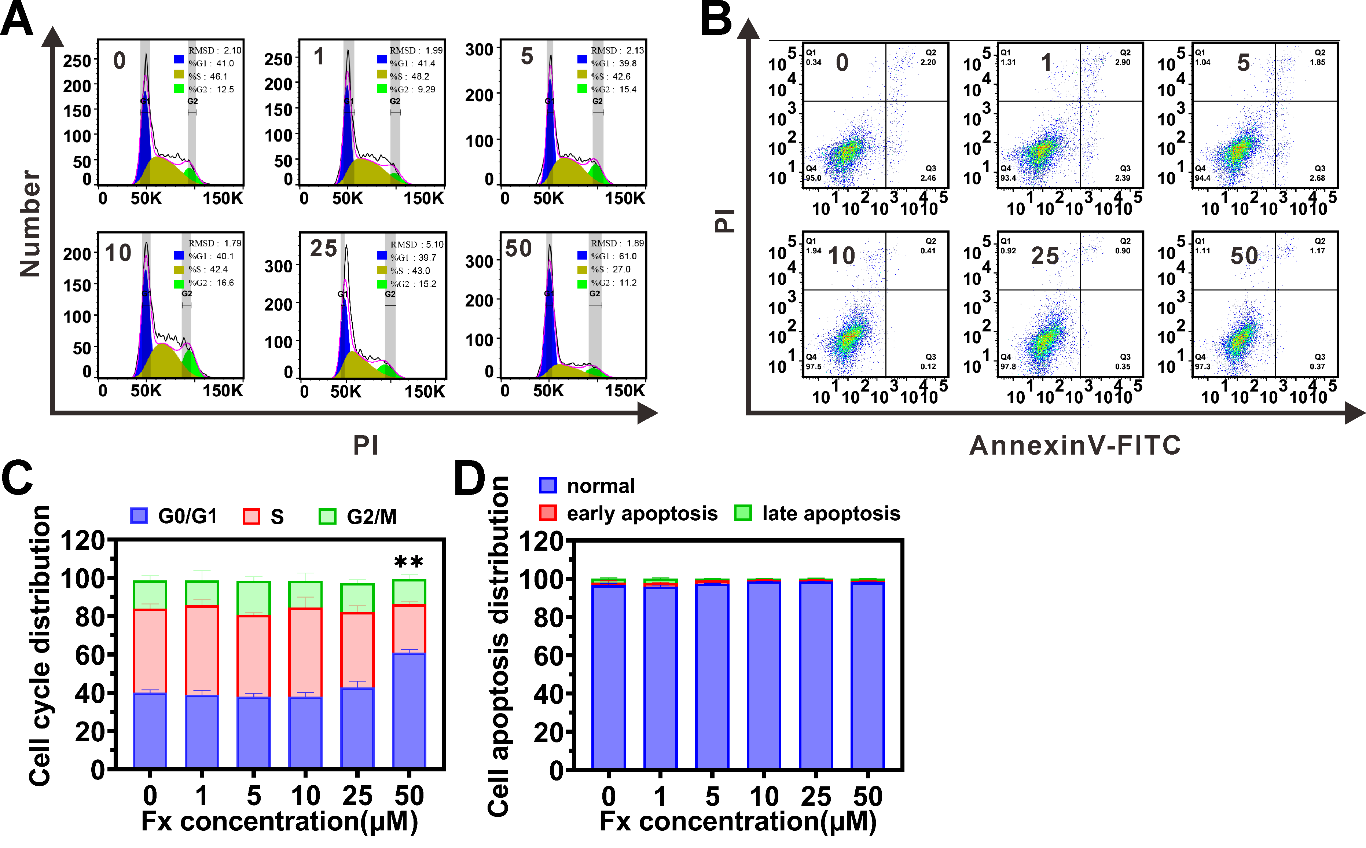


**Supplementary Figure 1.** **The effects of Fx on 4T1 cell cycle and apoptosis.** **A and B,** 4T1 cells were incubated with Fx for 24 h, cell cycle distribution (**A**) and apoptosis (**B**) were analyzed by flow cytometry. **C and D**, The distribution of cell cycle and apoptosis were shown as histogram. Data are presented as mean ± SD (n=3). ** indicate P < 0.01 vs Control.


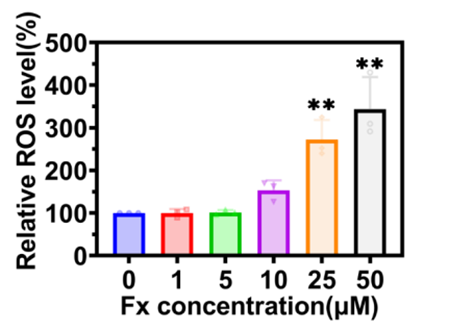


**Supplementary Figure 2.** Quantitative analysis of the intracellular ROS level in MCF-7 cells. Bars represent the mean ± SD (n=3); * indicate significant differences as compared with the control (**, p < 0.01).


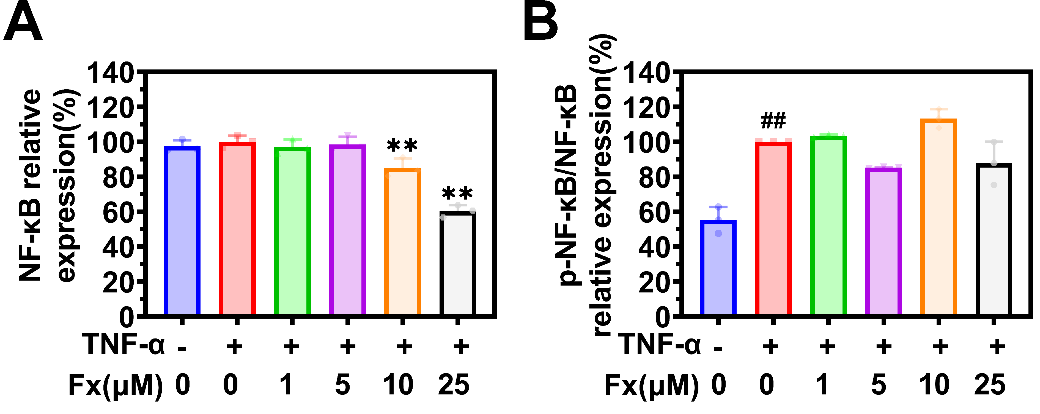


**Supplementary Figure 3.** Band intensity of NF-кB **(A)** and p-NF-кB/NF-кB **(B)** were quantified using Image Lab software and expressed as percentage of control (TNF-α only). Data are presented as mean ± SD (n=3); ** indicate *P* < 0.01 *vs* Control (TNF-α only); ^##^ indicate *P* < 0.01 *vs* Negative control (TNF-α free).


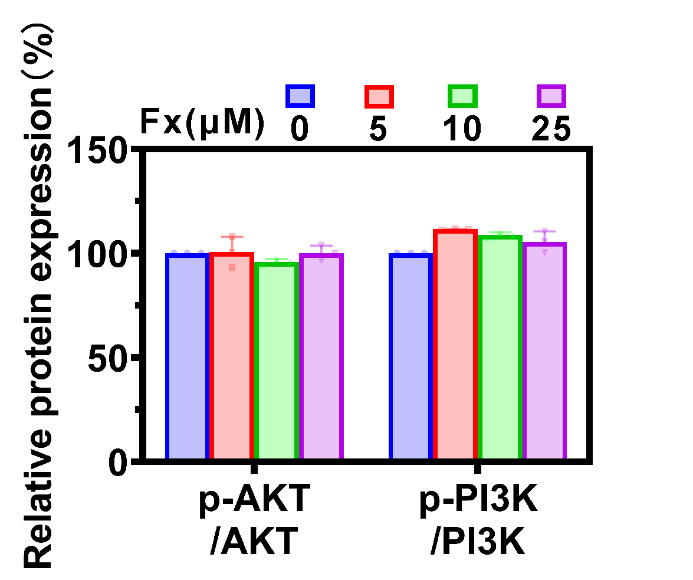


**Supplementary Figure 4**. Band intensity of pAKT/AKT and p-PI3K/PI3K were quantified using Image Lab software and expressed as percentage of control (TNF-α only). Data are presented as mean ± SD (n=3).


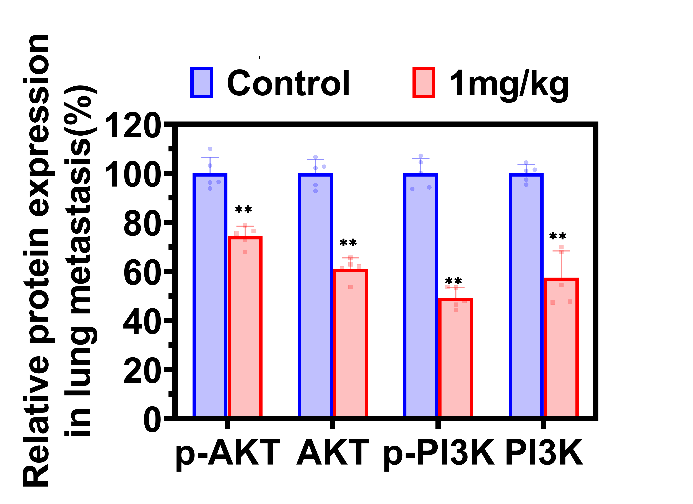


**Supplementary Figure 5.** Immunohistochemical quantification of positive staining in lung tumor metastases in BALB/c mice. Fx significantly inhibited the total and phosphorylation levels of AKT and PI3K proteins in lung tumor metastases.


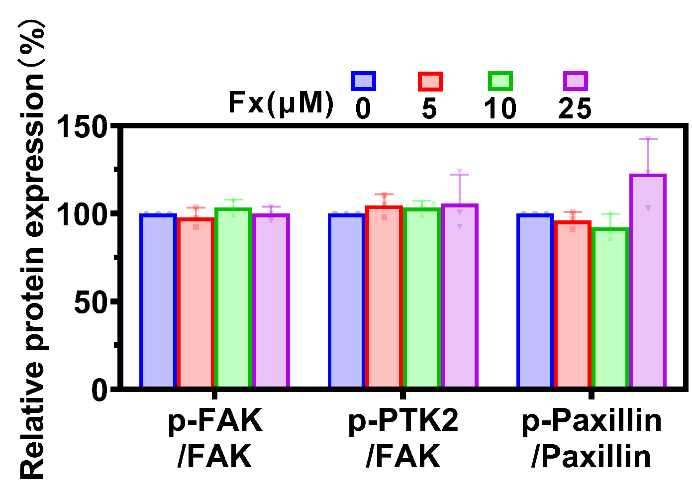


**Supplementary Figure 6**. Band intensity of p-FAK/FAK, p-PTK2/FAK and p-Paxillin/Paxillin were quantified using Image Lab software and expressed as percentage of control (TNF-α only). Data are presented as mean ± SD (n=3).


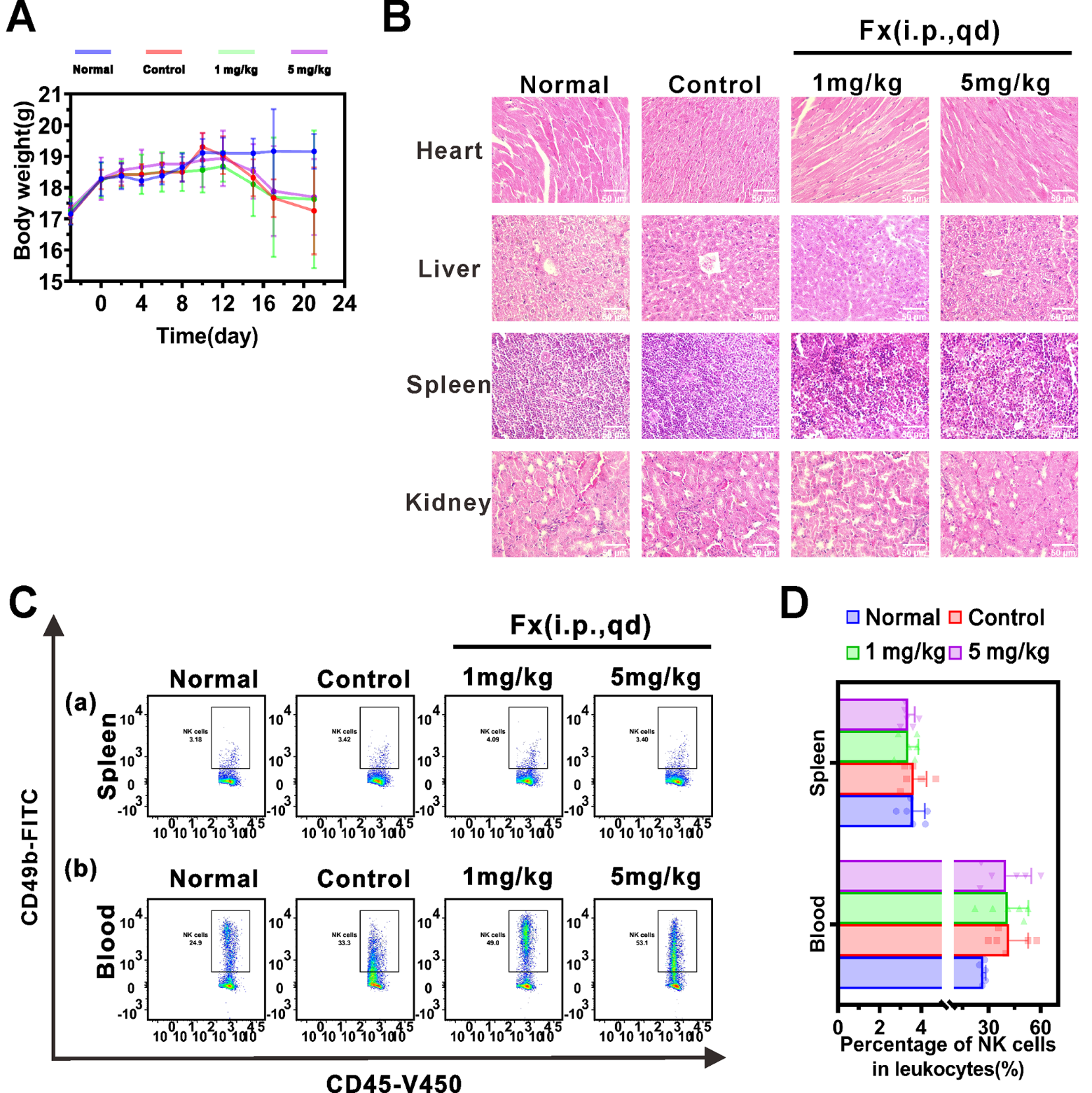


**Supplementary Figure 7.** **A**, Statistical data of the body weight of the mice treated with Fx. **B,** H&E staining of hearts, livers, spleens and kidneys of the mice treated with Fx. **C,** The percentage of natural killer cells (NK cells, CD45+CD49b+) in peripheral blood and spleen of mice was detected by flow cytometry. **D,** Quantitative analysis showed that the Fx did not significantly alter the percentage of NK cells in peripheral blood and spleen. Bars represent the mean ± SD (n=6).

# Data Availability Statement

# The original data generated for this study can be found in the Jianguoyun/Nutstore [https://www.jianguoyun.com/p/DS5wRlUQlefUChjjhcYEIAA].
